# Closely Located but Totally Distinct: Highly Contrasting Prokaryotic Diversity Patterns in Raised Bogs and Eutrophic Fens

**DOI:** 10.3390/microorganisms8040484

**Published:** 2020-03-29

**Authors:** Anastasia A. Ivanova, Alexey V. Beletsky, Andrey L. Rakitin, Vitaly V. Kadnikov, Dmitriy A. Philippov, Andrey V. Mardanov, Nikolai V. Ravin, Svetlana N. Dedysh

**Affiliations:** 1 Winogradsky Institute of Microbiology, Research Center of Biotechnology of the Russian Academy of Sciences, Moscow 119071, Russia; ivanovastasja@gmail.com (A.A.I.); dedysh@mail.ru (S.N.D.); 2 Institute of Bioengineering, Research Center of Biotechnology of the Russian Academy of Sciences, Moscow 119071, Russia; mortu@yandex.ru (A.V.B.); rakitin@biengi.ac.ru (A.L.R.); vkadnikov@bk.ru (V.V.K.); mardanov@biengi.ac.ru (A.V.M.); 3 Papanin Institute for Biology of Inland Waters, Russian Academy of Sciences, Borok 152742, Russia; philippov_d@mail.ru

**Keywords:** northern mires, raised bogs, eutrophic fens, high-throughput 16S rRNA gene sequencing, microbial diversity, *Acidobacteria*, *Verrucomicrobia*, *Chloroflexi*, *Planctomycetes*

## Abstract

Large areas in Northern Russia are covered by extensive mires, which represent a complex mosaic of ombrotrophic raised bogs, minerotrophic and eutrophic fens, all in a close proximity to each other. In this paper, we compared microbial diversity patterns in the surface peat layers of the neighbouring raised bogs and eutrophic fens that are located within two geographically remote mire sites in Vologda region using 16S rRNA gene sequencing. Regardless of location, the microbial communities in raised bogs were highly similar to each other but were clearly distinct from those in eutrophic fens. Bogs were dominated by the *Acidobacteria* (30%–40% of total 16S rRNA gene reads), which belong to the orders *Acidobacteriales* and *Bryobacterales*. Other bog-specific bacteria included the *Phycisphaera*-like group WD2101 and the families *Isosphaeraceae* and *Gemmataceae* of the *Planctomycetes*, orders *Opitutales* and *Pedosphaerales* of the *Verrucomicrobia* and a particular group of alphaproteobacteria within the *Rhizobiales*. In contrast, fens hosted *Anaerolineae*-affiliated *Chloroflexi*, *Vicinamibacteria*- and *Blastocatellia*-affiliated *Acidobacteria*, *Rokubacteria*, uncultivated group OM190 of the *Planctomycetes* and several groups of betaproteobacteria. The *Patescibacteria* were detected in both types of wetlands but their relative abundance was higher in fens. A number of key parameters that define the distribution of particular bacterial groups in mires were identified.

## 1. Introduction

Wetlands are one of the most biologically productive ecosystems and provide a wide range of essential ecosystem services, which are critical to human livelihoods and sustainable development [1]. They play a major role in the global water balance by receiving, storing and releasing water, regulating flows and supporting life. They are also recognized for their high nutrient recycling capacities and their prominent contribution to global greenhouse gas emissions. The global area of natural wetlands is about 5.3–5.7 × 10^6^ km^2^ [2,3]. Peat accumulating wetlands (peatlands) cover about 4.16 × 10^6^ km^2^ worldwide, with 80% of the peatland area situated in temperate-cold climates in the northern hemisphere, particularly in Russia, Canada and the USA [4]. These ecosystems serve as a persistent sink for atmospheric CO_2_ and a global terrestrial carbon store [5]. Peatlands are classified into various types based on vegetation and trophic status. Among those, raised bogs, which are fed solely by precipitation, and eutrophic fens, which are fed mainly by ground water, represent two most contrasting types of mires.

Bogs are peat forming wetlands with a high accumulation of organic material. They are highly acidic (pH values typically around 4.0), nutrient-poor by nature and are dominated by *Sphagnum* mosses. Microbial diversity in these peatlands was assessed in numerous cultivation-independent studies, which involved the use of fluorescence in situ hybridization, 16S rRNA gene sequence analysis, metagenomics and metatranscriptomics [6,7,8,9,10,11,12,13,14,15]. These habitats are usually dominated by members of the phyla *Acidobacteria* and *Proteobacteria*; other commonly present bacteria are affiliated with the *Verrucomicrobia*, *Actinobacteria* and *Planctomycetes*. A large proportion of the indigenous bacteria populations in acidic peat bogs is represented by as-yet-uncultivated organisms with unknown physiologies and metabolic potentials [16].

Fens are also peat forming wetlands but are less acidic and more nutrient-rich than bogs. The typical vegetation for this type of wetland is sedges and grasses. The microbial diversity in fens has received less research attention. Most studies have addressed microbial groups involved in methane cycling [6,17,18]. The comparative analysis of the microbial community structure at bog and fen sites in the Glacial Lake Agassiz Peatland of northwestern Minnesota revealed much higher microbial abundance and diversity in the fen than in the bog, as well as distinct diversity patterns [9]. The bog site was dominated by the *Acidobacteria*, while the *Firmicutes* dominated in the fen. Clear differences were observed also with regard to the archaeal community composition.

Russia is among the countries with the largest area of mires. The most recent estimates of the total mire area in European Russia indicate up to 15 million hectares [19]. Many large mire massifs in European North Russia represent a complex mosaic of ombrotrophic raised bogs, minerotrophic mires and eutrophic fens, all in a close proximity to each other. 

This study aimed at comparing microbial diversity patterns in the surface peat layers of the neighboring raised bogs and eutrophic fens that are located within two geographically remote mire sites in the Vologda region. As revealed by our analysis, the microbial communities in raised bogs were highly similar to each other but were clearly distinct from those in eutrophic fens. We also defined a number of environmental variables that determine distribution of several specific groups of peat-inhabiting microorganisms. 

## 2. Materials and Methods

### 2.1. Study Sites and Sampling Procedure

**Figure 1:**
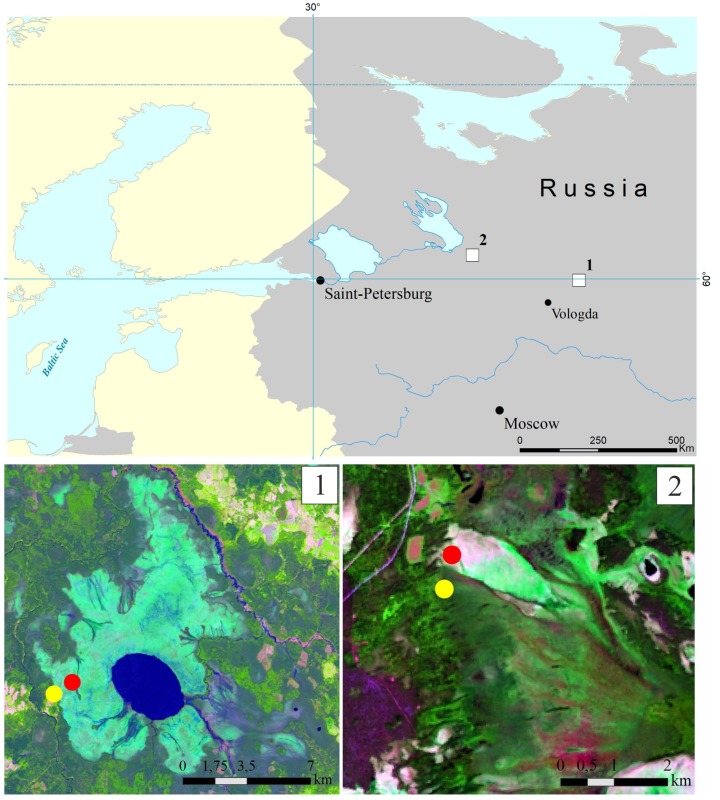
Location of the two study sites, the Shichengskoe (**1**) and Piyavochnoe (**2**) mires, on a map of European North Russia (upper panel). Sampling sites within the Shichengskoe (**1**) and Piyavochnoe (**2**) mires (bottom panel). Raised bogs and fens are indicated by red and yellow dots, respectively.

This study was performed in the Vologda region of European North Russia, within the zone of the middle taiga. Two large mire massifs, the Shichengskoe and Piyavochnoe mires, each displaying a high degree of spatial variability and comprising peatlands of different trophic status, were selected. The distance between these study sites was about 260 km (Figure 1).

The Shichengskoe mire is a large (15.9 km^2^) mire system, which was formed in the large glacial lake basin [20] (Figure 1). The central part of the mire is occupied by a shallow distrophic lake Shichengskoe (1060 ha). The distribution of ombrotrophic and minerotrophic areas in the mire system reflects the flows of gravitational and ground water. Significant area of the mire is occupied by the hummock–carpet raised bog, dominated by *Pinus*–shrublets–*Sphagnum* and *Eriophorum*–shrublets–*Sphagnum* associations. The eutrophic edges of the mire are forested with *Betula pubescens*, *Salix pentandra*, and *Alnus glutinosa* [20,21]. Two sampling sites, one located within the raised bog (59°56′56.9″ N, 41°16′59.4″ E) and another one within the eutrophic edge of the mire (59°56′31.6″ N, 41°15′53.5″ E), were chosen for the study (Figure 1, and in more details in Supplementary Figure S1).

The Piyavochnoe mire is a large (80 km^2^) mire complex composed of several raised bogs, aapa-mires and fen massifs, and a series of intra-mire primary lakes and mineral islands [22] (Figure 1). The hummock–carpet peat bog site selected for sampling purposes represented an unforested part of the mire with the uniform cover of *Sphagnum angustifolium* and *Eriophorum vaginatum* (60°46′29.8″ N, 36°49′35.4″ E). The eutrophic fen site was located on the forested edge of the mire (60°46′08.9″ N, 36°49′30.9″ E); the vegetation cover was composed of *Comarum palustre*, *Menyanthes trifoliate* and *Sphagnum warnstorfii* (Supplementary Figure S2). Detailed descriptions of plant communities in all sampling sites are given in Supplementary Table S1.

The sampling was performed on July 19 and 21, 2019. Three individual plots, on a distance of approximately 30–50 m from each other, were chosen within each study site for sampling purposes. The peat cores (30 × 30 × 30 cm; each sample of approximately 5 kg) were collected from the surface layer of the sampling plots and were transported to the laboratory in boxes containing ice packs. Each of the collected 12 peat cores was processed separately. The samples used for the analysis were taken from the upper, underlying vegetation cover peat layer, at a depth of 0–10 cm. The peat material from this layer was separated, homogenized and cut into small fragments (5–10 mm) with sterile scissors to prepare one composite sample for each of the cores. Three replicate samples were taken from each core and frozen at −20 °C for DNA extraction.

### 2.2. Chemical Analyses

Field measurements of pH, total dissolved solids and electrical conductivity were made using Combo HI 98129 analyzer (Hanna Instruments, Germany). The total organic carbon and total nitrogen contents were determined for the average sample from each plot using Vario MACRO Cube CN-analyser (Elementar Analysensysteme GmbH, Germany). Concentrations of Fe, Ca, Mg and P were determined by means of inductively coupled plasma mass spectrometry (ICP-MS Agilent 7500a, Agilent, Santa Clara, CA, USA), while the concentration of sulfates was determined using the Dionex ICS-2000 Ion Chromatography System (Dionex, Sunnyvale, CA, USA).

### 2.3. DNA Extraction and Sequencing Procedure

Soil samples were frozen in liquid nitrogen and ground using a porcelain mortar and pestle. Total DNA was isolated from 0.25 g of soil samples using DNeasy PowerSoil Kit (Qiagen, Hilden, Germany) according to the manufacturer’s instructions. The V3–V4 variable region of the prokaryotic 16S rRNA genes was obtained by PCR with primers 341F (5’- CCTAYGGGDBGCWSCAG) and 806R (5’- GGACTACNVGGGTHTCTAAT) [23]. PCR fragments were barcoded using Nextera XT Index Kit v2 (Illumina, USA). The PCR fragments were purified using Agencourt AMPure Beads (Beckman Coulter, Brea, CA, USA) and quantitated using Qubit dsDNA HS Assay Kit (Invitrogen, Carlsbad, CA, USA). Then all the amplicons were pooled together in equal moral amounts and sequenced on the Illumina MiSeq instrument (2 × 300 nt reads). One of the sequencing reactions from the triplicate set of preparations for plot II of the fen Piyavochnoe failed and, therefore, only 2 replicates were obtained for this sample. Paired overlapping reads were merged using FLASH [24].

### 2.4. Bioinformatic Analyses

The pool of 16S rRNA gene sequences was analyzed with *QIIME 2* v.2019.10 (https://qiime2.org) [25]. DADA*2* plugin was used for sequence quality control, denoising and chimera filtering [26]. Operational Taxonomic Units (OTUs) were clustered applying VSEARCH plugin [27] with open-reference function using Silva v. 132 database [28,29] with 97% identity. Taxonomy assignment was performed using BLAST against Silva v. 132 database with 97% identity. The alpha-diversity indices were calculated using the core-metrics-phylogenetic method implemented in QIIME v. 2.2019.10. UniFrac and Principle Coordinate Analysis (PCoA) were also carried out in QIIME2 via q2-diversity function [30,31,32]. The significance of weighted and unweighted UniFrac matrices were calculated with Permanova test [33]. Microbial community composition and abundance were visualized using GraPhlAn via Galaxy platform (http://huttenhower.sph.harvard.edu/galaxy/) [34].

MicrobiomeSeq v. 0.1 (https://github.com/umerijaz/microbiomeSeq) R package was used to calculate Pearson correlation coefficients between abundances of the taxonomic groups and environmental factors. The significance of the correlation was tested by calculating the *p*-values, adjusted for multiple comparisons using Benjamin and Hochberg method in MicrobiomeSeq v.0.1.

### 2.5. Nucleotide Sequence Accession Number

The raw data generated from 16S rRNA gene sequencing were deposited in Sequence Read Archive (SRA) under the accession numbers SRR11280489 -SRR11280524, available via BioProject PRJNA610704. 

## 3. Results

### 3.1. Peat Chemistry in Bogs and Fens

The peat samples collected from bog and fen sites displayed a number of key differences with regard to their chemical composition (Table 1). The bog waters were more acidic (pH 3.7–4.3) than those in fen sites (pH 6.4–6.9). The values of peat water conductivity in fens (394–408 and 130–225 µS cm^−1^ in Shichengskoe and Piyavochnoe fens, respectively) were far above those in bogs (58–72 and 63–64 µS cm^−1^). While the total organic carbon contents in peat collected from these two types of peatlands were similar, the fens contained twice as much total nitrogen as the raised bogs. The concentrations of Ca, Mg, Fe and P in peat from fens by far exceeded those in peat from bogs (Table 1).

### 3.2. Sequencing Statistics and Alpha-Diversity Metrics

A total of 850,783 partial (average length, ~440 bp) 16S rRNA gene sequences were obtained from the peat samples collected from Shichengskoe and Piyavochnoe mire massifs (Table 2). Of these, 420,342 reads were retained after quality filtering, denoising and removing chimeras. Overall, the microbial community composition was more diverse in the two fens (average Shannon index 8.14 ± 0.11 and Pielou evenness 0.85 ± 0.01, mean ± SE) then in the corresponding raised bogs (average Shannon index 6.92 ± 0.10 and Pielou evenness 0.89 ± 0.00) (Table 2). 

The number of species-level OTUs determined at 97% sequence identity ranged between 186 and 391 in the bogs and between 384 and 965 in the fens. 

**Figure 2:**
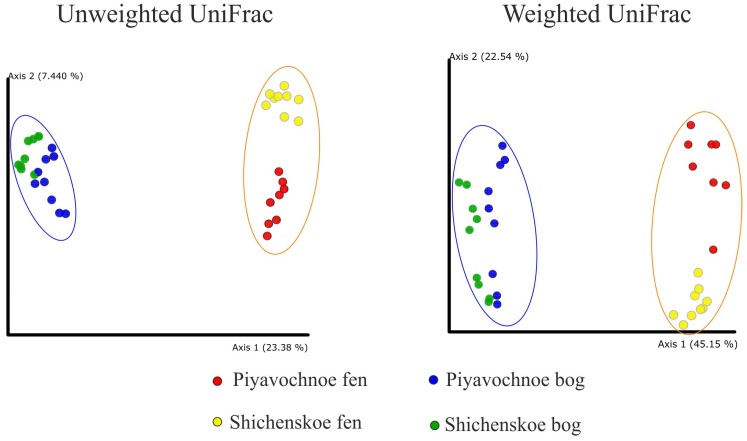
Comparison of the microbial community composition in peat samples examined in this study by principle coordinate analyses (PCoA). PCoA plot is based on the weighted and unweighted UniFrac distance of the sequencing dataset. The significance of differences between the microbial community compositions in bogs and fens is confirmed with *p* ≤ 0.001 for both weighted and unweighted UniFrac matrices.

As revealed by the UniFrac analysis and a further Permanova test, the microbial assemblages in the two geographically remote raised bogs were highly similar to each other but were significantly (*p* ≤ 0.001) different to those in eutrophic fens (Figure 2).

### 3.3. Microbial Diversity Patterns at the Phylum Level

**Figure 3:**
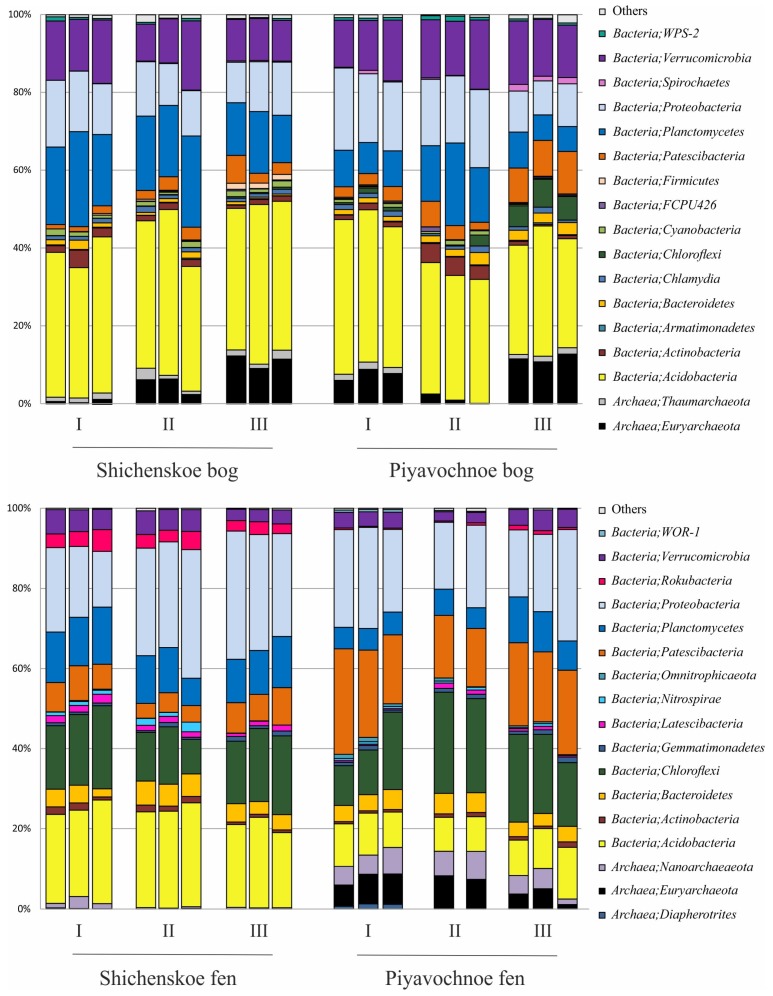
Bacteria and Archaea community composition in two raised bogs (upper panel) and two fens (lower panel) according to the results of Illumina 16S rRNA gene sequencing. The composition is displayed at the phylum level. All replicates are shown.

The pools of reads retrieved from the examined peat samples were dominated by 16S rRNA gene sequences of bacterial origin (Figure 3). The relative abundance of archaeal 16S rRNA gene reads ranged from 0.2% to 14.4% of all sequences. 

Archaeal populations in both raised bogs were represented by members of the *Euryarchaeota* and *Taumarchaeota*. The fen Shichengskoe was characterized by a very low relative abundance of archaea, which were nearly exclusively represented by members of the *Nanoarchaeota*. The latter group of archaea was also present in the fen Piyavochnoe (mean ± SE, 4.9 ± 0.6% of total reads) along with *Euryarchaeota* (5.5 ± 0.8%) and *Diapherotrites* (0.4 ± 0.2%) (Figure 3).

Bacterial communities in the two raised bogs were dominated by the *Acidobacteria* (37.6 ± 1.1% and 33.5 ± 1.4% of total reads retrieved from the Shichengskoe and Piyavochnoe bogs, respectively). Other major groups were the *Proteobacteria* (13.2 ± 0.7% and 15.6 ± 1.5%), *Planctomycetes* (18.3 ± 1.4% and 10.9 ± 1.6%), and *Verrucomicrobia* (12.8 ± 1.0% and 14.6 ± 0.6%) (Figure 3). Less abundant groups of bacteria, which were detected in both raised bogs included the *Patescibacteria* (2.9 ± 0.6% and 5.6 ± 1.1%), *Chloroflexi* (0.2 ± 0.1% and 2.7 ± 0.9 %), *Actinobacteria* (1.9 ± 0.4% and 2.2 ± 0.6%), *Bacteroidetes* (1.1 ± 0.2% and 2.1 ± 0.3%), *Chlamydiae* (1.1 ± 0.1% and 1.1 ± 0.1%), *Cyanobacteria* (1.2 ± 0.1% and 0.6 ± 0.1%), *Spirochaetes* (0.2 ± 0.0% and 0.7 ± 0.2%), *Firmicutes* (0.6 ± 0.2% and 0.2 ± 0.0%) and WPS-2 (0.5 ± 0.1% and 0.7 ± 0.1%). At the phylum level, the microbial community compositions in the two studied raised bogs were highly similar to each other (Figure 3).

In contrast to raised bogs, the microbial assemblages in both fens were dominated by members of the *Proteobacteria* (24.8 ± 2.1% and 20.9 ± 1.4% of total reads retrieved from the Shichengskoe and Piyavochnoe bogs, respectively) and *Chloroflexi* (15.8 ± 1.3% and 17.9 ± 1.9%). The third numerically abundant group of reads in the fens Shichengskoe and Piyavochnoe were the *Acidobacteria* (22.6 ± 0.8% and 9.6 ± 0.5%) and *Patescibacteria* (6.4 ± 0.6% and 18.9 ± 1.3%). The 16S rRNA gene sequences affiliated with the *Planctomycetes* (11.4 ± 0.7% and 7.0 ± 0.8%) and *Bacteroidetes* (4.4 ± 0.4% and 4.1 ± 0.2%) were retrieved from both fens. Several minor groups of bacteria that were detected in fens but were absent from raised bogs included the *Rokubacteria* (3.5 ± 0.3% and 0.5 ± 0.1%), *Latescibacteria* (1.5 ± 0.1% and 0.7 ± 0.1%), and *Nitrospirae* (0.9 ± 0.3% and 0.5 ± 0.1%).

### 3.4. Bacterial Groups Characteristic for Specific Types of Mires

**Figure 4:**
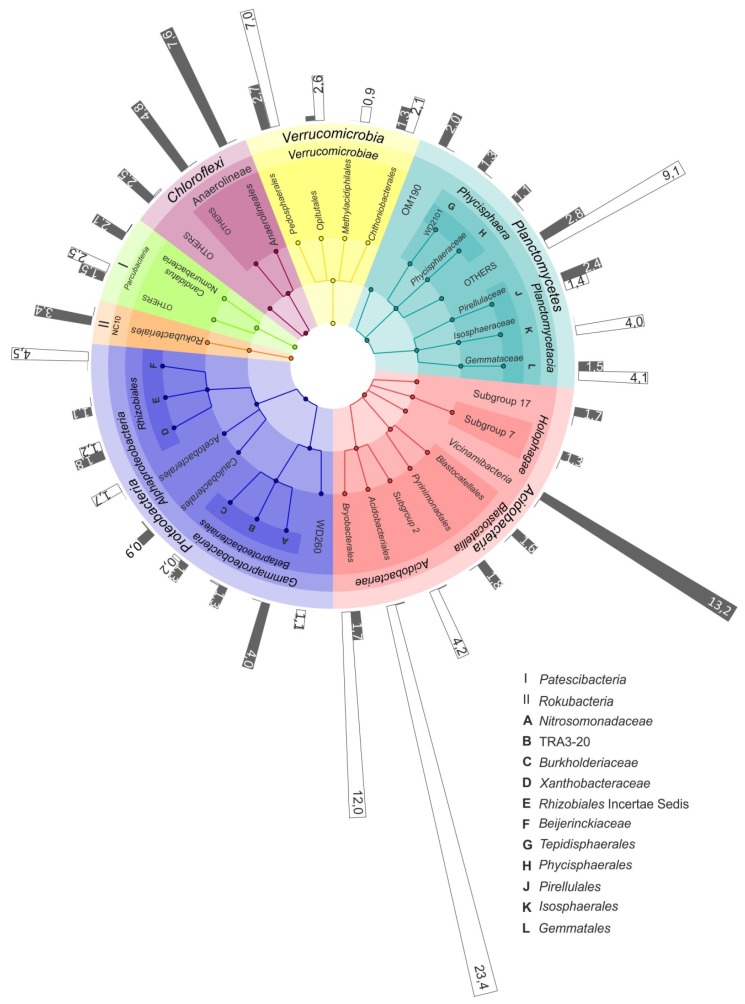
The most representative microbial groups in the bog and fen sites of the Shichengskoe mire. The outermost circle shows the relative abundance of specific microbial group in the raised bog (white bars) and in the fen (grey bars). The colored nodes from inner ring to outer ring indicate taxonomic groups from phylum to family level.

The difference between the microbial community compositions in raised bogs and fens became even more pronounced when the analysis was performed at the sub-phylum level. Thus, a high relative abundance of the *Acidobacteria* was detected both in the bog and the fen of the mire Shichengskoe (Figure 3). In the bog, however, this phylum was represented by members of the class *Acidobacteriia*, i.e., the orders *Acidobacteriales*, *Bryobacterales* and as-yet-uncultivated Subdivision 2 (SD2) (Figure 4). In contrast, most acidobacterial 16S rRNA gene sequences retrieved from the fen Shichengskoe were affiliated with the class *Vicinamibacteria*, although members of the *Blastocatellia* as well as SDs 7 and 17 were also present. A highly similar pattern in the *Acidobacteria* distribution was observed in peat samples collected from the Piyavochnoe mire (Supplementary Figure S3).

The pools of proteobacterial reads retrieved from the two raised bogs were dominated by *Alphaproteobacteria*- affiliated 16S rRNA gene sequences, with the most abundant group of sequences from as-yet-uncultivated members of the order *Rhizobiales*. The latter, by contrast, were only poorly represented in the two fens, where members of the *Betaproteobacteria* became one of the most abundant proteobacterial groups (Figure 4; Supplementary Figure S3).

Some habitat-specific diversity patterns were also observed for the *Planctomycetes*. Thus, *Isosphaeraceae*-like planctomycetes were found exclusively in raised bogs, while members of the as-yet-uncultivated group OM190 were present only in eutrophic fens (Figure 4; Supplementary Figure S3). The most abundant group of planctomycetes in the bogs, i.e., the *Phycisphaera*-like group WD2101, however, was present at low abundances in eutrophic fens as well. A similar distribution patterns was also characteristic of several groups within the *Verrucomicrobia*, i.e., the *Pedosphaerales*, *Chtoniobacteriales* and *Opitutales*. *Methylacidophilales*-like verrucomicrobia were detected exclusively in raised bogs.

Members of the candidate division “*Patescibacteria*” were most abundant in the fens, with the highest relative abundance and diversity detected in the Piyavochnoe fen (Supplementary Figure S3). Representatives of another candidate division, the “Rokubacteria”, were found exclusively in the fens (Figure 3 and Figure 4), with the highest relative abundance (3.4% of all reads) detected in the Shichengskoe fen.

### 3.5. Most Abundant Habitat-Specific OTUs

**Figure 5:**
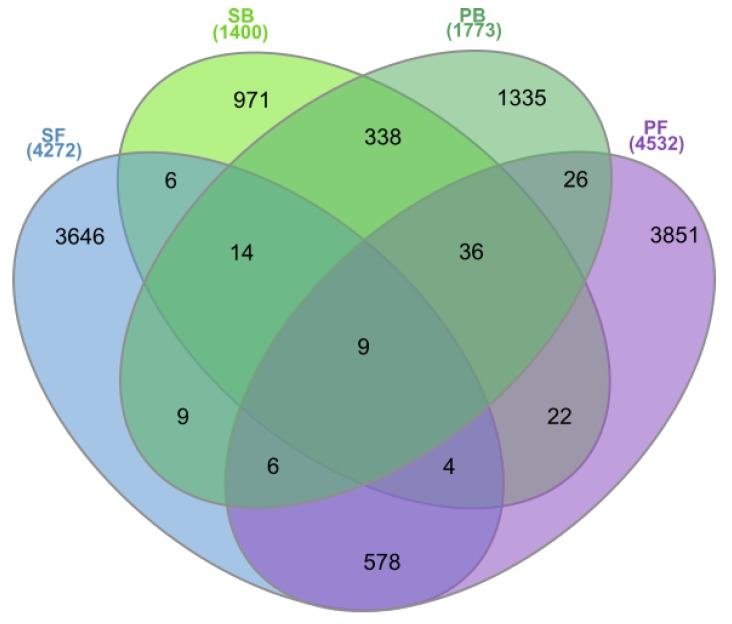
The Venn diagram showing the number of Operational Taxonomic Units (OTUs) shared by different peatland sites: Shichengskoe bog (SB), Shichengskoe fen (SF), Piyavochnoe bog (PB), and Piyavochnoe fen (PF).

The pools of OTUs shared between the two geographically remote bog sites and between the two fen sites included 338 and 578 OTUs, respectively (Figure 5). By contrast, the neighboring bog and fen sites in Shichengskoe mire had only 33 common OTUs, while 77 common OTUs were identified for the bog and fen sites in the mire Piyavochnoe. Only nine OTUs were shared between all four peatlands examined in this study.

The list of most abundant OTUs (≥ 0.7% of all reads retrieved from the corresponding peatland type), which were specific for either bogs or fens, is given in Table 3. One half of bog-specific OTUs was represented by members of the class *Acidobacteriia*, orders *Acidobacteriales* and *Bryobacterales* as well as SD2. Only some of these OTUs could be classified at the genus level, as representing the genera *Bryobacter*, *Occallatibacter*, and *Candidatus* Solibacter. Another group of abundant bog-specific OTUs was affiliated with the *Planctomycetes* and belonged exclusively to *Phycisphaera*-like WD2101 soil group. *Verrucomicrobia*-affiliated OTUs in bogs were represented by members of the *Opitutales* and *Pedosphaerales*. Interestingly, one of the most abundant bog-specific OTUs affiliated with the *Patescibacteria* and belonged to the *Parcubacteria*.

Since the microbial communities in the two fens were more distinct than those in the bogs (Figure 2), the list of fen-specific OTUs with the relative abundance ≥ 0.7% of all reads included six records only (Table 3). These included *Anaerolineaceae*- and KD4-96 group-related *Chloroflexi*, WD2101 group-related *Planctomycetes*, alphaproteobacteria of the family *Hyphomonadaceae,* acidobacteria of the class *Vicinamibacteria* and *Candidatus* Nomurabacteria of the *Patescibacteria*.

### 3.6. Correlation between Peat Properties and Abundance of Microbial Groups

**Figure 6:**
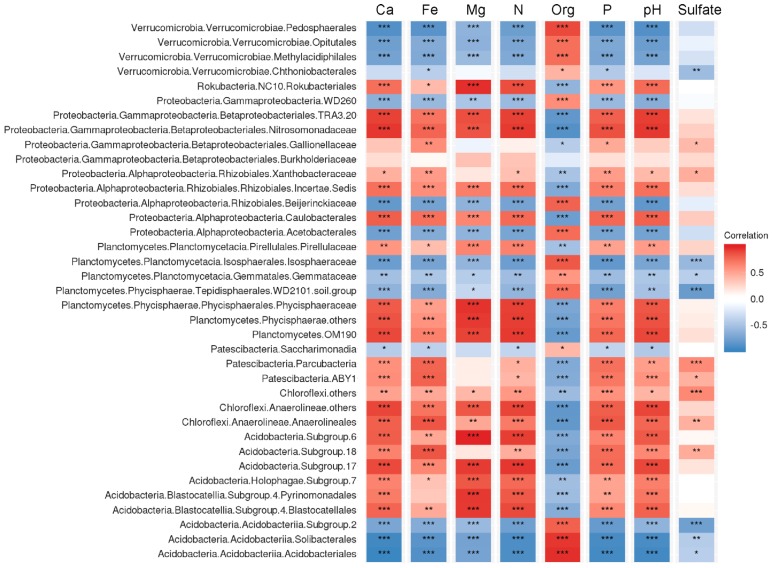
The correlation matrix based on Pearson’s correlation analysis between peat properties and abundance of microbial groups. The colour of rectangle represents the strength of the correlation. Correlations passing the significant level (*p* < 0.05, *p* < 0.01, *p* < 0.001) were marked by one, two and three asterisks, respectively. Ca, Fe, Mg, P, - concentrations of Ca, Fe, Mg, P. Org, total organics content (%). N, total nitrogen content (%).

Correlation analysis performed for the number of key bacterial groups in peatlands showed that many of those were highly related with the variation of peat properties (Figure 6). Relative abundances of the *Acidobacteriia,* several groups within the *Verrucomicrobia* (*Pedosphaerales*, *Opitutales*, *Methylacidophilales*) and *Planctomycetes* (*Isosphaeraceae*, WD2101) were positively correlated with total organic carbon content (TOC) but negatively correlated with pH and total nitrogen content (TN). The opposite correlation pattern (negative correlation with TOC but positive correlation with pH and TN) was characteristic of the *Vicinamibacteria,* uncultivated group of planctomycetes OM190, *Anaerolineales, Caulobacterales* and uncultivated group within the *Rhizobiales*. Notably, a strong positive correlation with Fe availability was observed in members of the *Parcubacteria*, *Anaerolineales* and SD18 of the *Acidobacteria*. The relative abundances of *Rokubacteria* were positively correlated with TN as well as Ca and Mg availability.

## 4. Discussion

As shown in our study, the microbial assemblages in two raised bogs located at a distance of 260 km were highly similar to each other but were clearly distinct from those in two eutrophic fens, which are located in a close proximity (at a distance of several hundred meters) to the bogs. From our research, the trophic status and geochemical characteristics of these two different types of peatlands were the major factors that shaped the microbial community composition in these ecosystems.

Independently of the geographic location, the diversity patterns obtained in different studies for acidic and nutrient-poor *Sphagnum*-dominated peat bogs are highly reproducible [6,7,8,9,10,11,14,17,35]. Surface peat layers in these peatlands are commonly dominated with *Acidobacteria*, although *Alphaproteobacteria*, *Planctomycetes* and *Verrucomicrobia* are also present in a high abundance. Diversity analysis at the phylum level, however, is clearly insufficient for identifying a spectrum of bog-specific microorganisms. Thus, most acidobacteria that are commonly detected in bogs are affiliated with one particular class of this phylum, i.e., the *Acidobacteriia* [36]. The latter accommodates aerobic and facultatively anaerobic, acidophilic or acidotolerant, mesophilic and psychrotolerant, chemoheterotrophic bacteria, which utilize various sugars and polysaccharides, and possess a number of hydrolytic capabilities including the abilities to degrade cellulose and chitin [37,38]. The most abundant bog-specific OTU determined in our study belonged to the as-yet-uncultivated group within the order *Acidobacteriales* and displayed highest similarity to the environmental clone sequence (GenBank accession No. FR720610) retrieved from a *Sphagnum* peat bog in Yaroslavl region, European North Russia [10]. Several other abundant bog-specific OTUs listed in Table 3 are affiliated with the genus *Bryobacter* [39]. Acidobacteria of this genus were isolated from boreal peat bogs and are capable of utilizing galacturonic and glucuronic acids, which are released during decomposition of *Sphagnum* moss. One particular gap in our knowledge of bog-inhabiting acidobacteria is represented by Subdivision 2 (SD2) of this phylum, which also falls within the taxonomic range of the class *Acidobacteriia* but does not include characterized representatives. SD2 acidobacteria are often detected in *Sphagnum*-dominated wetlands [11,40] but, so far, have resisted all cultivation efforts. According to the results of our correlation analysis (Figure 6), SD2 acidobacteria are most likely phenotypically similar to members of the *Acidobacteriales* and *Bryobacterales*. The reasons behind our failure to culture these bacteria remain unknown.

Among the bog-specific populations of *Planctomycetes*, *Phycisphaera*-like WD2101 soil group deserves particular attention. This group was named after the environmental 16S rRNA gene sequence WD2101 (GenBank accession No. AJ292687) retrieved by Nogales et al. [41] from an acidic polychlorinated biphenyl-polluted soil near Wittenberg, Germany. Members of this group have been detected by cultivation-independent approaches in a wide variety of peatlands [42]. At present, WD2101 soil group is classified within the order *Tepidisphaerales* of the class *Phycisphaerae.* The only characterized representative of this order is the moderately thermophilic, polysaccharide-degrading planctomycete from terrestrial hot springs, *Tepidisphaera mucosa* [43]. *Tepidisphaera mucosa* grows between 20 and 56 °C and in the pH range 4.5–8.5. The 16S rRNA gene sequence similarity between *Tepidisphaera mucosa* and the corresponding gene fragments retrieved from peat is low (~90%), suggesting that bog-inhabiting members of this order belong to as-yet-undescribed family and may possess different temperature and pH adaptations. As indicated by our correlation analysis (Figure 6), these planctomycetes are acidophilic, oligotrophic heterotrophs, which do not depend on availability of mineral nutrients.

Another bog-specific bacterial group of interest is represented by *Verrucomicrobia*-affiliated 16S rRNA gene reads which, according to the classification system implemented in Silva v.132 database, are classified as belonging to the order “*Methylacidophilales”*. These sequences were detected in several molecular diversity studies of acidic peat bogs [11,44]. The “*Methylacidophilaceae”* is a candidate family that accommodates extremely acidophilic methanotrophic bacteria, which grow at pH < 5 [45]. These extremophilic methanotrophs were found in several environments over a wide temperature range but seem to be restricted to geothermally influenced habitats [46]. The sequences retrieved from peatlands display only a low similarity (84–87%) to 16S rRNA gene sequences of currently described verrucomicrobial methanotrophs. The occurrence of verrucomicrobial methanotrophs in acidic peatlands, therefore, remains an open question.

In comparison to the results of diversity analyses in peat bogs, the reports on microbial community compositions in fens show more variability. The latter, apparently, is largely dependent on pH value and concentrations of individual nutrients, such as mineral nitrogen, sulfate, Fe and others. In addition to the *Proteobacteria*, which are always present as a major bacterial group, different fens may contain *Firmicutes* [9], *Chloroflexi* [8], *Actinobacteria* [17] or *Acidobacteria* ([17]; this study) as the second numerically abundant group. Members of the *Bacteroidetes* are also common members of the microbial community in fens. The relative abundance of these bacteria in peatlands is largely determined by the availability of mineral nitrogen [10].

The two fens examined in our study also hosted a large diversity of bacteria from several candidate phyla, such as *Patescibacteria*, *Latescibacteria*, *Rokubacteria* and WOR-1. Thus, a surprisingly high relative abundance of *Patescibacteria* (15–30% of total 16S rRNA gene reads) was detected in the fen Piyavochnoe (Figure 3). Since an ectosymbiotic lifestyle has been suggested for this group of as-yet-uncultivated bacteria with small streamlined genomes [47], their potential host(s) should also have been present in this fen as one of the major bacterial groups. High relative abundances of several understudied phyla with no cultured representatives make eutrophic fens an attractive object of further metagenome-based insights into the metabolic capabilities of these elusive bacteria, which are expected to uncover their functional potential and to explain their wide distribution in peatlands.

## Figures and Tables

**Table 1:** Characteristics of the sampling sites

Characteristics	Shichengskoe Mire		Piyavochnoe Mire	
Sampling Site	Raised Bog	Fen	Raised Bog	Fen
Coordinates	59°56′56″ N, 41°16′59″ E	59°56′31″ N, 41°15′53″ E	60°46′29″ N, 36°49′35″ E	60°46′08″ N, 36°49′30″ E
Sampling date	19.08.2019	19.08.2019	21.08.2019	21.08.2019
Water characteristics				
water level depth, cm	4 7	8 13	5 8	11 13
pH	4.3	7.4	3.7	6.9
T, °C	12.2–13.3	11.1–11.5	12.8–13.6	12.9–13.3
Total dissolved solids, ppm	29–36	197–204	31–32	65–113
Electrical conductivity, µS/cm	58–72	394–408	63–64	130–225
Peat characteristics:				
Total organic carbon (%)	88.5	73.6	85.1	71.6
N total (%)	0.605	2.31	0.923	1.65
Sulfate (mg/L)	172	202	220	222
Fe (ppm)	343	9387	1347	16344
Ca (ppm)	3522	29834	4190	27373
Mg (ppm)	634	2575	682	1078
P (ppm)	614	1179	791	1305
Plant community	*Eriophorum vaginatum* –*Sphagnum angustifolium*	*Equisetum palustre* –*Sphagnum warnstorfii*	*Eriophorum vaginatum* –*Sphagnum angustifolium*	*Comarum palustre* –*Menyanthes trifoliata*–*Sphagnum warnstorfii*
Vegetation coverage, %	97–98	95–97	98–99	97–98

**Table 2:** Sequencing statistics and alpha-diversity metrics.

Sampling Site	Sample ID	Technical Replicates	Input Reads	Filtered Reads	Denoised Reads	Non-Chimeric Reads	Input/Non-Chimeric (%)	Diversity Indices		
								Shannon	Observed OTUs	Pielou Evenness
Shichenskoe raised bog	**I**	**1**	16,569	13,986	11,328	7911	47.75	6.26	234	0.79
		**2**	13,368	11,311	8653	7871	58.88	7.06	263	0.88
		**3**	9981	8448	6162	5837	58.48	6.78	219	0.87
	**II**	**1**	36,199	30,577	24,854	19,354	53.47	7.23	343	0.86
		**2**	26,855	22,556	18,200	15,028	55.96	6.88	293	0.84
		**3**	21,574	18,317	14,728	12,698	58.86	7.24	299	0.88
	**III**	**1**	25,592	21,468	17,926	14,643	57.22	6.70	269	0.83
		**2**	15,806	13,431	11,061	9140	57.83	6.60	218	0.85
		**3**	22,249	18,550	15,202	12,560	56.45	6.77	246	0.85
Shichenskoe fen	**I**	**1**	66,173	55,426	34,436	33,475	50.59	9.25	965	0.93
		**2**	41,081	34,587	19,400	18,869	45.93	8.72	757	0.91
		**3**	22,977	19,098	10,130	9904	43.10	8.16	538	0.90
	**II**	**1**	13,255	10,914	4391	4391	33.13	7.67	384	0.89
		**2**	18,070	15,079	7765	7744	42.86	8.12	530	0.90
		**3**	11,726	9796	3941	3855	32.88	7.58	379	0.89
	**III**	**1**	17,321	14,565	7189	7114	41.07	7.88	460	0.89
		**2**	14,215	11,974	5838	5795	40.77	7.73	428	0.88
		**3**	19,799	16,609	8456	8332	42.08	7.84	457	0.89
Piyavochnoe fen	**I**	**1**	59,217	49,585	30,626	29,298	49.48	8.80	865	0.90
		**2**	29,471	24,831	14,240	13,779	46.75	8.33	651	0.89
		**3**	30,310	25,511	14,449	14,029	46.29	8.17	569	0.89
	**II**	**1**	15,285	12,807	5039	5030	32.91	7.64	440	0.87
		**2**	27,096	22,699	11,467	11,253	41.53	8.22	632	0.88
	**III**	**1**	21,514	17,694	8561	8548	39.73	7.97	484	0.89
		**2**	22,856	19,147	9301	9132	39.95	8.06	542	0.89
		**3**	26,518	22,124	11,157	10,862	40.96	8.16	558	0.89
Piyavochnoe raised bog	**I**	**1**	23,968	20,170	15,768	13,598	56.73	7.21	329	0.86
		**2**	13,966	11,754	8665	7783	55.73	6.77	251	0.85
		**3**	30,399	24,950	19,350	16,556	54.46	7.31	343	0.87
	**II**	**1**	28,820	24,120	17,711	15,710	54.51	7.39	355	0.87
		**2**	29,935	25,098	18,802	16,641	55.59	7.57	374	0.89
		**3**	33,092	27,498	20,233	18,084	54.65	7.58	391	0.88
	**III**	**1**	19,036	16,003	12,938	11,136	58.50	6.69	252	0.84
		**2**	11,608	9639	7182	6435	55.44	6.27	186	0.83
		**3**	14,882	12,387	9439	7947	53.40	6.32	219	0.81

**Table 3:** Most abundant OTUs common for microbial communities of either peat bogs or fens.

	OTU	%	Closest Silva Match	Sequence Identity (%)	Taxonomy
Raised Bogs	1	2.0	FR720610	97.8	*Acidobacteria; Acidobacteriia; Acidobacteriales*
	2	1.9	HQ598818	96.5	*Acidobacteria; Acidobacteriia; Acidobacteriales; Acidobacteriaceae* (SD 1)
	3	1.5	FJ625320	99.7	*Acidobacteria; Acidobacteriia; Bryobacterales; Bryobacteraceae* (SD 3)*; Bryobacter*
	4	1.5	HM445984	90.2	*Patescibacteria; Parcubacteria*
	5	1.5	EF516015	98.3	*Acidobacteria; Acidobacteriia; Acidobacteriales*
	6	1.5	HQ598778	99.3	
	7	1.4	GU127746	93.8	*Planctomycetes; Phycisphaerae; Tepidisphaerales;* WD2101 soil group
	8	1.4	EF173346	98.5	*Acidobacteria; Acidobacteriia; Acidobacteriales; Acidobacteriaceae* (SD 1)*; Occallatibacter*
	9	1.3	AY792285	99.0	*Proteobacteria; Alphaproteobacteria; Rhizobiales; Beijerinckiaceae*
	10	1.2	CZKI01000047	95.0	*Verrucomicrobia; Verrucomicrobiae; Opitutales; Opitutaceae*
	11	1.0	GU983329	97.0	*Acidobacteria; Acidobacteriia; Bryobacterales; Bryobacteraceae* (SD 3)*; Candidatus* Solibacter
	**12**	1.0	AY792311	96.0	*Verrucomicrobia; Verrucomicrobiae; Pedosphaerales; Pedosphaeraceae*
	**13**	1.0	GU727715	97.5	*Acidobacteria; Acidobacteriia; Bryobacterales; Bryobacteraceae* (SD 3)*; Bryobacter*
	**14**	0.9	GQ402663	95.5	*Planctomycetes; Phycisphaerae; Tepidisphaerales;* WD2101 soil group
	**15**	0.8	AY963300	94.8	
	**16**	0.8	AM162437	97.8	*Proteobacteria; Alphaproteobacteria; Rhizobiales; Beijerinckiaceae; Roseiarcus*
	**17**	0.8	GU127795	97.5	*Acidobacteria; Acidobacteriia; Acidobacteriales*
	**18**	0.8	HM445277	98.0	*Proteobacteria; Gammaproteobacteria;* WD260
	**19**	0.7	EU150204	97.5	*Acidobacteria; Acidobacteriia;* SD 2
	**20**	0.7	HQ597923	96.3	*Acidobacteria; Acidobacteriia; Bryobacterales;Bryobacteraceae* (SD 3)*; Candidatus* Solibacter
	**21**	0.7	JN023510	96.8	
Fens	**22**	1.3	AB630565	99.5	*Chloroflexi; Anaerolineae; Anaerolineales; Anaerolineaceae*
	**23**	1.1	JQ311867	92.8	*Planctomycetes; Phycisphaerae; Tepidisphaerales;* WD2101 soil group
	**24**	0.9	LN570440	94.5	*Proteobacteria; Alphaproteobacteria; Caulbacterales; Hyphomonadaceae*
	**25**	0.8	HM062099	97.5	*Vicinamibacteria*
	**26**	0.8	FQ659415	86.2	*Patescibacteria; Parcubacteria; Ca*. Nomurabacteria
	**27**	0.7	EF019369	96.8	*Chloroflexi*; KD4-96

## Supplementary Materials

The following are available online at https://www.mdpi.com/2076-2607/8/4/484/s1. Figure S1: The mire Shichengskoe; Figure S2: The mire Piyavochnoe; Figure S3: The most representative microbial groups in the bog and fen sites of the Piyavochnoe mire; Table S1: Plant community composition of the sampling sites.
