# Supplementary material for: Closely Located but Totally Distinct: Highly Contrasting Prokaryotic Diversity Patterns in Raised Bogs and Eutrophic Fens
Source: Microorganisms. 2020 Mar 29;8(4):484. doi: 10.3390/microorganisms8040484 (PMC7232223; doi:10.3390/microorganisms8040484)
Supplement: Supplementary file 1 [file microorganisms-08-00484-s001.pdf]

Supplementary Table S1. Plant community composition of the sampling sites.

Plant species	Shichengskoe mire		Piyavochnoe mire	
	Raised bog	Fen	Raised bog	Fen
Total number of species (range in individual sampling plots):	6 (4–6)	25 (12–15)	4 (3–4)	15 (10–12)
**DWARF SHRUBS**				
Andromeda polifolia L.	+	–	–	–
Oxycoccus palustris Pers.	+	+	+	+
Chamaedaphne calyculata (L.) Moench	+	–	+	–
Rubus arcticus L.	–	+	–	–
Rubus chamaemorus L.	+	–	–	–
**SEDGE PLANTS**				
Carex dioica L.	–	+	–	+
Carex flava L.	–	+	–	–
Carex lasiocarpa Ehrh.	–	+	–	–
Eriophorum latifolium Hoppe	–	+	–	–
Eriophorum vaginatum L.	+	–	+	–
Trichophorum alpinum (L.) Pers.	–	+	–	–
**GRAMINEOUS PLANTS**				
Poa alpigena (Blytt) Lindm.	–	–	–	+
**HERBS**				
Angelica sylvestris L.	–	+	–	–
Cardamine pratensis L.	–	–	–	–
Comarum palustre L.	–	–	–	+
Crepis paludosa (L.) Moench	–	+	–	–
Drosera rotundifolia L.	–	+	–	–
Equisetum fluviatile L.	–	+	–	–
Equisetum palustre L.	–	+	–	–
Filipendula ulmaria ssp. *denudata* (J. Presl & C. Presl) Hayek	–	+	–	–
Galium palustre L.	–	+	–	–
Galium trifidum L.				+
Galium uliginosum L.	–	+	–	–
Geum rivale L.	–	+	–	–
Gymnadenia conopsea (L.) R. Br.	–	–	–	+
Menyanthes trifoliata L.	–	+	–	+
Potentilla erecta (L.) Raeusch.	–	+	–	–
Rumex acetosa ssp. *fontano-paludosus* (Kalela) Hyl.	–	+	–	+
Thyselium palustre (L.) Raf.	–	+	–	–
Viola epipsila Ledeb.	–	+	–	–
**MOSSES**				
Aulacomnium palustre (Hedw.) Schwägr.	–	+	–	+
Campylium stellatum (Hedw.) C.E.O. Jensen	–	+	–	+
Helodium blandowii (F.Weber & D.Mohr) Warnst.	–	–	–	+

Sphagnum angustifolium (C.E.O. Jensen ex Russow) C.E.O. Jensen	+	–	+	+
Sphagnum warnstorfii Russow	–	+	–	+
Straminergon stramineum (Dicks. ex Brid.) Hedenäs	–	–	–	+
Tomentypnum nitens (Hedw.) Loeske	–	+	–	–

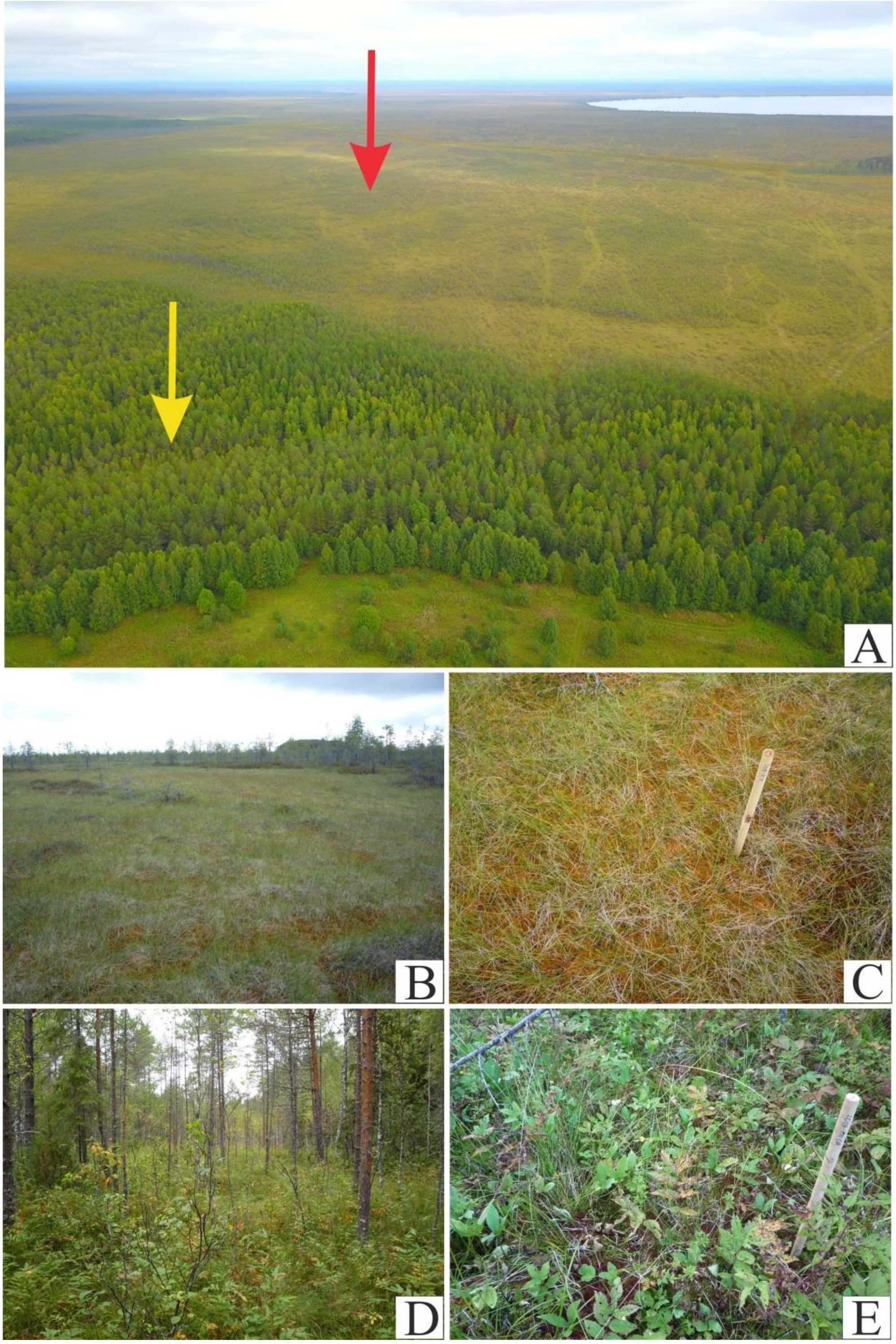

Supplementary Figure S1. The mire Shichengskoe.

(**A**) General overview of the mire Shichengskoe. Locations of the study sites corresponding to the raised bog and fen are shown by red and yellow arrows, respectively. (B) Overview of the raised bog and (C) the indigenous plant community. (D) Image of the fen and (E) the indigenous plant community.

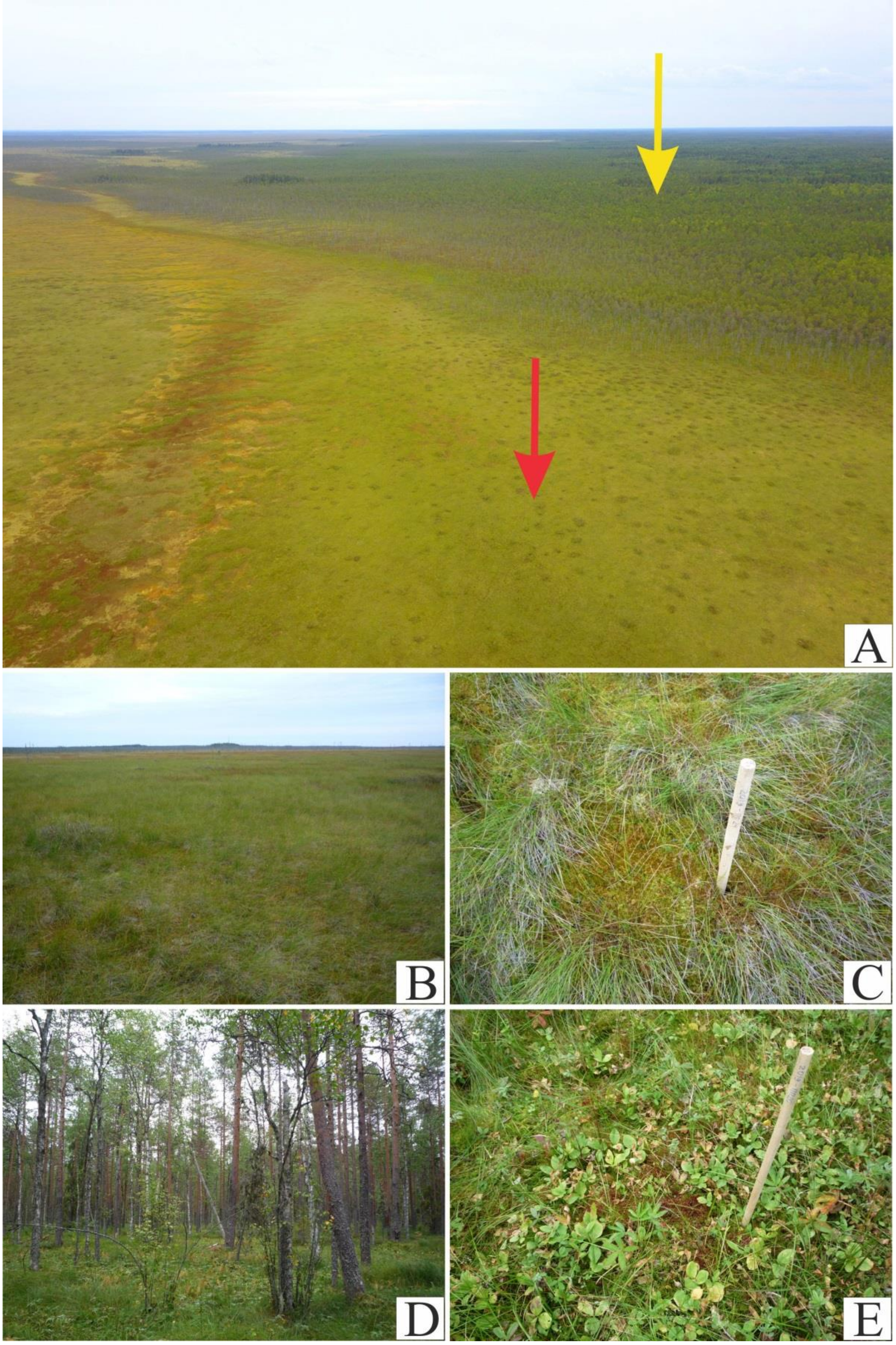

Supplementary Figure S2. The mire Piyavochnoe.

(**A**) General overview of the mire Piyavochnoe. Locations of the study sites corresponding to the raised bog and fen are shown by red and yellow arrows, respectively. (**B**) Overview of the raised bog and (**C**) the indigenous plant community. (**D**) Image of the fen and (**E**) the indigenous plant community.

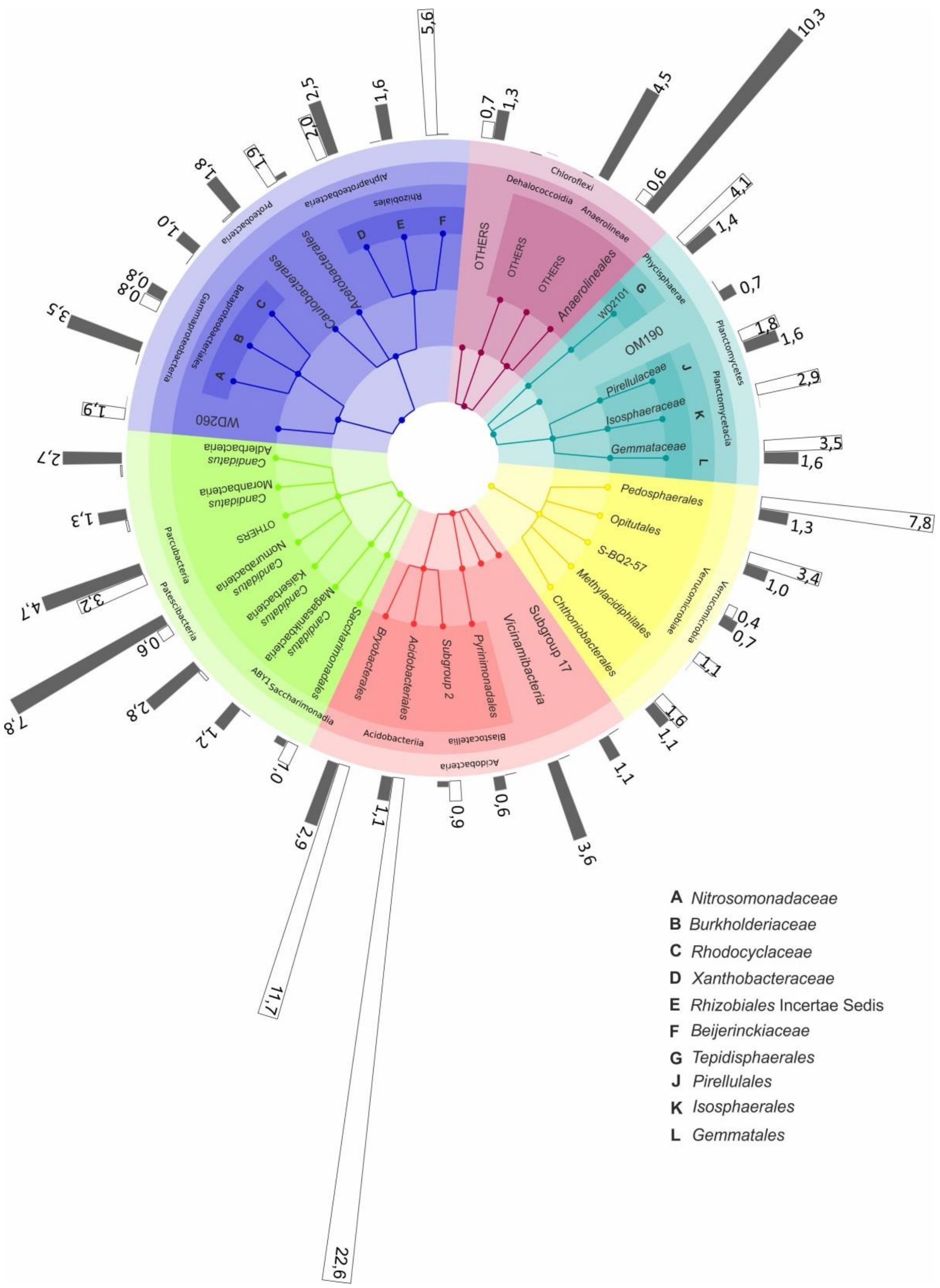

Supplementary Figure S3. The most representative microbial groups in the bog and fen sites of the Piyavochnoe mire.

The outermost circle shows the relative abundance of specific microbial group in the raised bog (white bars) and in the fen (grey bars). The colored nodes from inner ring to outer ring indicate taxonomic groups from phylum to family level.
